# Screening of Neurodevelopmental and Psychiatric Disorders in School-Aged Children from Sahrawi Refugee Camp: A Cross-Sectional Observational Study

**DOI:** 10.3390/jcm14062080

**Published:** 2025-03-18

**Authors:** Ilaria Accorinti, Linda Bonezzi, Gianluca Sesso, Simona Pipino, Francesca Pignatelli, Alessandra De Angelis, Annarita Milone, Roberta Battini

**Affiliations:** 1 Department of Neuroscience, IRCCS Stella Maris Foundation, 56128 Pisa, Italy; ilaria.accorinti@fsm.unipi.it (I.A.);; 2 Department of Clinical and Experimental Medicine, University of Pisa, 56126 Pisa, Italy; 3 School of Medicine, Faculty of Medicine and Biomedical Sciences, The University of Queensland, Brisbane, QLD 4072, Australia; 4 Social and Affective Neuroscience Group, Molecular Mind Lab, IMT School for Advanced Studies Lucca, 55100 Lucca, Italy; 5 Residency School of Pediatrics, University of Rome “Tor Vergata”, Via Montpellier 1, 00133 Rome, Italy; 6 Faculty of Medicine and Surgery, University Cattolica del Sacro Cuore, 00168 Rome, Italy

**Keywords:** community psychiatry, school mental health, child mental health

## Abstract

**Background/Objectives**: The prevalence of neurodevelopmental and psychiatric disorders in children is a growing concern in developed countries. However, data from low- and middle-income countries (LMICs) remain scarce. The objective of this study was to ascertain the prevalence of such conditions in the school-aged children residing in Sahrawi refugee camps. In particular, the objective was to develop a bespoke screening instrument for the collection of epidemiological data and the examination of the impact of these disorders on academic performance and school life. **Methods**: A cross-sectional observational study was conducted in 13 primary schools within Sahrawi refugee camps, with a target sample size of 3425 children aged 7–14. The Strengths and Difficulties Questionnaire (SDQ) was administered to teachers to identify any neurodevelopmental issues. **Results**: A total of 74 (2.2% [95% CI: 1.7–2.7%]) of the 3425 children displayed positive SDQ results for neurodevelopmental or psychiatric difficulties. The most frequently identified issues were emotional and conduct problems, which often co-occurred. Boys exhibited higher hyperactivity rates than girls. **Conclusions**: This study’s findings revealed significant neurodevelopmental and psychiatric challenges in Sahrawi children, with implications for their academic and social development. The results emphasize the necessity of enhancing teacher training and mental health interventions to facilitate early identification and support.

## 1. Introduction

In recent decades, there has been a growing awareness of the importance of addressing the mental health needs of children and adolescents. Indeed, the prevalence of neurodevelopmental disorders has increased in Western countries [1,2]. Meanwhile, epidemiological studies in low- and middle-income countries (LMICs) are scarce, and the current lack of standardization of the collection and reporting of data on neurodevelopmental conditions presents a significant challenge to the interpretation of prevalence estimates. A limited number of studies conducted in LMICs have reported an overall prevalence of 7% of attention deficit hyperactivity disorder (ADHD) [3], 0–28% of depression, and 8–27% of anxiety [4]. These figures are in stark contrast to the prevalence rates observed in the United States, which stand at 11.4%, 4%, and 10% respectively [5]. Prevalence studies on autism spectrum disorder (ASD) in Arab countries appear to be particularly limited; preliminary reports from Saudi Arabia and Jordan suggest prevalences of 1:10,000 and 1:50, respectively [6]. These are considerably lower than the prevalence rate of 1:36 reported in Western countries [7].

There is a considerable disparity in mental health policies across countries, with low-income countries bearing the burden of this inequity [8]. The current focus on mental health in Africa is limited to adults and severe disabilities. For children and adolescents, clinical attention is primarily focused on stress-related mental health issues, such as acute stress disorders and post-traumatic stress disorders (PTSDs), which often arise in the aftermath of wars, migration, and human rights violations [9]. However, the latest literature reveals a lack of data concerning other non-trauma-related neurodevelopmental and psychiatric conditions in children and adolescents residing as permanent refugees in LMICs.

One such population facing these challenges is the Sahrawi community, which has been residing in Algerian refugee camps since the mid-1970s due to ongoing territorial and political disputes. As of 2018, approximately 173,600 Sahrawis, including 65,000 minors, lived across five provinces (Wilayas) [10]. The children of this community, now in their second and third generation as refugees, have not directly experienced conflict but continue to face significant challenges in their daily lives. These include limited access to essential resources such as healthcare, education, and economic opportunities. From a demographic and epidemiological standpoint, the Sahrawi refugee population displays characteristics that are typical of a relatively closed community [11], which may influence health and developmental outcomes.

Despite the challenges of life in refugee camps, the Sahrawi have established an education system that is both structured and widespread. Primary education is compulsory for children aged 6 to 11, while secondary education is also mandatory, although it is not compulsory. Future teachers receive minimal training on supporting children with developmental disabilities, such as ADHD, ASD, and learning disorders. The school committee, comprising a pediatrician, psychologist, and nurse, monitors students but is limited in providing specialized care. Special education centers serve children with severe disabilities, offering curricula aligned with their cognitive impairment in the first years, while adolescents above the age of 12 receive occupational therapy and learn essential life skills (e.g., cooking, shopping, and sewing). However, the absence of specialized physicians means that many children with cognitive or motor impairments are not diagnosed or treated adequately.

Beyond education, the broader living conditions in the Sahrawi refugee camps present substantial public health challenges. The camps, located in arid desert regions, rely heavily on international humanitarian aid for food, healthcare, and water supply. According to the World Food Programme [12], severe food insecurity affects 7% of the population, while 57% experience moderate food insecurity. Over the past decade, acute and chronic malnutrition rates have increased, reaching 10.5% and 29% in children between 6 months and 5 years old. Access to clean water remains a significant issue, with over 50% of the population relying on water delivered by trucks, leading to high costs and concerns about water quality [10].

The healthcare system within the camps consists of a central hospital, five regional hospitals (one in each Wilaya), and over 20 primary healthcare centers. These facilities offer basic medical care, but the lack of specialized services means that individuals requiring advanced treatment must seek care in Algeria or, in some cases, travel to Europe through international humanitarian programs [13]. The limited availability of healthcare professionals further exacerbates the situation, making the diagnosis and treatment of chronic conditions, including neurodevelopmental and psychiatric disorders, particularly challenging [14].

Providing adequate educational support for children with developmental disabilities is not merely a matter of fairness; it also has long-term implications for the Sahrawi community as a whole. Children who are supported in their educational journey are more likely to stay in school, achieve better academic outcomes, and develop the skills they need to contribute to their communities as adults [15]. Without it, children are at higher risk of dropping out of school, which can lead to negative outcomes such as unemployment, poverty, and social marginalization. Nevertheless, the current system lacks the necessary resources and expertise to fully support children with developmental disabilities. To improve outcomes, there is a crucial need to expand the actual teacher training programs, reinforce the role of the school committee, and implement targeted interventions for children with conditions such as ADHD, ASD, and learning disabilities. This will enable the Sahrawi education system to better serve its students and ensure that all children, regardless of their abilities, can succeed. This is not only a matter of individual well-being but also a crucial investment in the long-term health and prosperity of the Sahrawi community.

### Aims of This Study

Currently, no epidemiological data exist on neurodevelopmental disorders among Sahrawi children and adolescents, which presents a significant challenge in assessing their treatment and rehabilitation needs. The unique situation of the Sahrawi population, characterized by a prolonged state of refugee status, a stable yet isolated environment with minimal interaction with other populations, and a reliance on UN resources, presents a compelling research opportunity. Furthermore, the education system represents a significant point of strength within this community, offering an invaluable foundation for future intervention.

The principal objectives of the present study were to (1) develop a screening tool tailored to the Sahrawi population to gather epidemiological data on major neurodevelopmental and psychiatric conditions in primary school children and (2) analyze how these disorders affect school life, including attendance, relationships, and academic performance.

The screening project also targeted teachers, with the additional objective of raising the local awareness of neurodevelopmental disorders and designing future interventions involving teachers and school principals. These interventions are intended to enhance the recognition of such conditions and mitigate their impact on children’s school attendance and overall well-being, as proposed in similar initiatives [15,16].

## 2. Method

This cross-sectional observational study was conducted in primary schools within Sahrawi refugee camps in Tindouf. This study followed the “Strengthening the Reporting of Observational Studies in Epidemiology” (STROBE) guidelines for cohort studies (Supplement 1) [17]. It was approved by the Institutional Review Board of the IRCCS Stella Maris Foundation and accepted by the SADR Government (approval code Najmatan-2023-RASD) as part of a humanitarian mission organized by the non-profit volunteer organization “Medicina e Assistenza ai Margini”.

The research team comprised two child psychiatrists, two pediatricians, a developmental psychologist, a speech therapist, and an Italian–Arabic interpreter.

### 2.1. Study Sample

With the approval of the local government, this study was conducted in 13 randomly selected primary schools to minimize selection bias and ensure representativeness. The schools included 3 of the 11 located in Smara (2382 students), 3 of the 6 in Auserd (2136 students), and 7 of the 10 in Laayoune (6405 students). This study was conducted between 25 February and 10 March 2023. Eligible participants were pupils in the third, fourth, and fifth grades, provided their teachers were available to complete both study phases. First and second graders were excluded to avoid false positives, as the lack of compulsory preschool education could result in increased adaptation difficulties, such as challenges with separating from parents, following rules, and maintaining attention. All data were collected anonymously, and teacher participation was voluntary, based on verbal informed consent obtained following a brief explanation of this study’s purpose and process. 

### 2.2. Methodology

**Figure 1:**
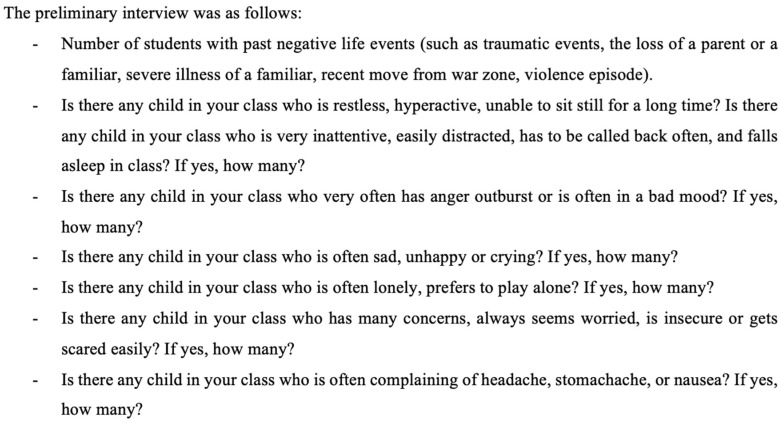
Preliminary interview administered to teachers.

The initial phase of this research process involved the participation of teaching staff in a preliminary interview (Figure 1). This was conducted by two psychiatrists, with the simultaneous provision of translation by an interpreter. The interview, designed specifically for the purposes of this study, aimed to gather general information about the students and consisted of two sections. The first section gathered epidemiological data, including class-specific information, sex distribution, age range, school attendance in the morning or afternoon sessions, and the number who had left the school in the previous year. Furthermore, considering the ongoing political instability in the region and the influx of recently displaced children from conflict zones, as well as the possibility of military involvement of fathers, a question on traumatic life events was incorporated. The second part of the preliminary interview concerned the identification of potential indicators of major neurodevelopmental and psychiatric conditions, utilizing questions adapted from the SDQ. The objective of this preliminary interview was to identify a smaller cohort of students for further assessment in the subsequent phase of this study.

In accordance with the responses provided by teachers, students could manifest symptoms indicative of primary neurodevelopmental and psychiatric disorders, which were categorized as follows: “hyperactivity/attention deficits”, “anger outburst” (meaning the difficulties in emotion regulation and consequent behavioral problems), “sad/unhappy”, “lonely” (meaning children with social relationship difficulties; this term was used as it was the definition given to these children by the Sahrawi teachers), “insecure/easily scared”, and “frequent complaining of pain (somatic complaints)”.

In the second phase of this study, the Strengths and Difficulties Questionnaire (SDQ) was then administered as a screening tool for developmental difficulties. Each teacher underwent a brief training session on instrument compilation, then completed the SDQ for each child identified in the preliminary interview. The SDQ was administered to the teachers individually in Arabic via the Jotform–Form, Sign & Survey App (version 2.9.74) on electronic devices (tablets and smartphones). In the event of any ambiguity, clarifications were promptly provided. The SDQ was selected for its simplicity, rapid administration, standardization in both Italian and Arabic, and extensive use in prevalence studies worldwide, rendering it appropriate for our study [16,18,19,20,21,22,23,24].

### 2.3. Instruments

The SDQ is a brief screening questionnaire designed for children aged 2–17 years, administered either to teachers, parents, or through self-reporting (aged 11–17). The SDQ comprises 25 attributes—some positive and others negative—organized into five 5-item scales: emotional symptoms, conduct problems, hyperactivity/inattention, peer relationship problems, and prosocial behavior. On the first four scales, an elevated score is indicative of a more severe clinical condition. Conversely, a lower score on the prosocial behavior scale signifies a greater degree of clinical difficulty. Scales from one to four added together give a total difficulty score, based on 20 total items. The total difficulty scores is derived from the first four scales, totaling 20 items, with scores categorized using the newer 4-band categorization. The categories “very high” and “high” or “very low” and “low” (on the Prosocial Scale) were considered positive indicators. The extended version of the SDQ includes an impact supplement, which assesses whether the respondent considers the child to have a problem and explores further aspects such as chronicity, distress, social impairment, and burden on others [25]. The additional data allow researchers and clinicians to better differentiate clinical difficulties beyond the total symptom score. In particular, an impact score of 2 or more is the most discriminating impact-based predictor of clinical status [26].

For this study, only the teacher version of the SDQ was administered, as parents were often unavailable due to various reasons, such as inadequate academic skills or work commitments.

### 2.4. Ethical Considerations

This study was conducted in accordance with the ethical principles outlined in the Declaration of Helsinki (1975, revised 2013) and the WHO International Ethical Guidelines for Epidemiological Studies.

As the Sahrawi refugee camps operate under the independent administration of the SADR, there is no formally established institutional review board. Ethical oversight for this study was therefore provided by the relevant governmental authorities, in accordance with national and international ethical guidelines.

This study received formal approval from the Sahrawi Arab Democratic Republic (SADR), which serves as the highest regulatory body overseeing research ethics within the refugee camps.

Given the cultural and linguistic context of the Sahrawi refugee population, verbal consent was deemed the most appropriate and ethical method of obtaining participation. This approach aligns with WHO guidelines on informed consent in low-literacy populations.

### 2.5. Statistical Analysis

The statistical analyses were conducted using Rstudio software (version R 4.0.2, Posit, Boston, MA, USA). The results are summarized as mean or median (with standard deviation, SD) for continuous variables and as frequency (%) for categorical variables. A *p*-value of less than 0.05 (two-tailed) was considered statistically significant, and a 95% confidence interval (95% CI) was calculated for all estimates.

Univariate analysis with Student’s *t*-test was used to detect significant differences (*p*-value < 0.05) in continuous variables between independent groups. Student’s *t*-test was performed only after confirming normal distributions using Shapiro’s test and homogeneity of variances by Levene’s test; otherwise, the nonparametric Mann–Whitney U test was used when normality could not be established. Analysis of variance (ANOVA) was applied after normality assumption was verified through the Shapiro test and used to assess significant differences (*p*-value < 0.05) in clinical variables with continuous distribution between demographic groups. If the normality assumption was not satisfied, the Kruskal–Wallis rank test was used. A Tukey’s post hoc test was used whenever ANOVA led to a statistically significant result to identify significant comparisons between variables.

## 3. Results

### 3.1. Epidemiologic Results

**Figure 2:**
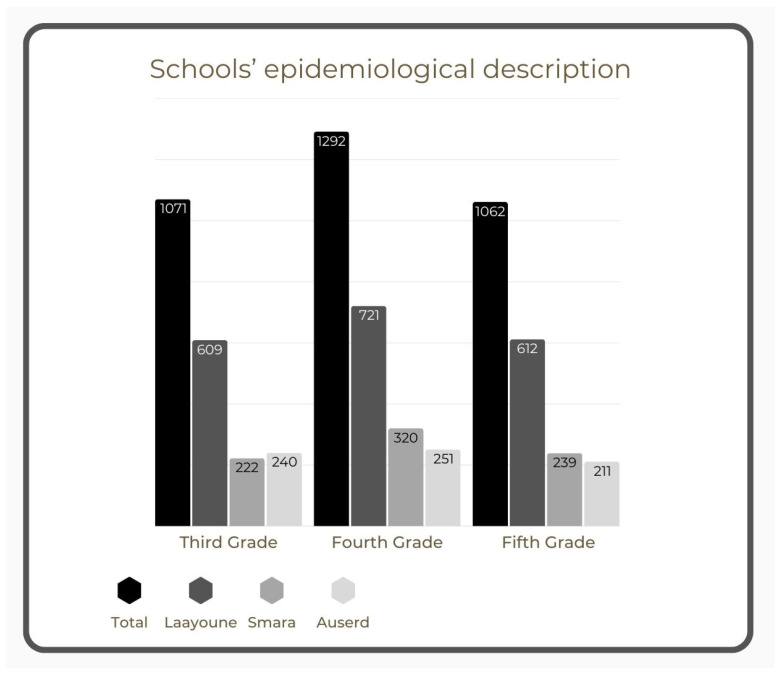
Description of the epidemiological characteristics of the educational institution in question.

The total number of students enrolled was 10,923; 7498 pupils did not meet the eligibility criteria due to either the attended class or teacher availability. The total number of children finally included in this study was 3425; 1071 children in third grade (26 teachers), 1292 in fourth grade (34 teachers), and 1062 in fifth grade (28 teachers). Figure 2 provides a description of the epidemiological characteristics of the educational institution in question. Based on the Wilayas, 781 students living in Smara, 702 students in Auserd, and 1942 students in Laayoune participated in this study. The number of eligible pupils varied between schools, from 177 to 525, with an average of 263.5 children per school; the average number of pupils per class was 35.31. The sex distribution varied between classes with an average rate of 49.72 ± 8.53% boys per class. Age (7–14 years) was relatively evenly distributed across the entire study population with a mean age of 10.15 ± 1.06 years.

### 3.2. Preliminary Interview

The data showed a dropout rate during the last year of 0.52% [95% CI: 0.31–0.82%] (18 children out of 3443). Comparing the three Wilayas and the different grades, there was a significant difference only in number of dropouts among the three grades (*p*-value = 0.005), specifically between the third (0%) and the fifth (12 out of 1074; 1.12% [95% CI: 0.58–1.94%])-grade classes (*p*-value = 0.016).

The number of students with negative early life events was 64 (1.87% [95% CI: 1.44–2.38%]). The results were compared among the three Wilayas, showing a significant difference (*p*-value = 0.01); the post hoc test showed a significant difference between the Wilaya of Laayoune (2.73% [95% CI: 2.05–3.55%]) and the Wilaya of Smara (0.38% [95% CI: 0.07–1.11%]) (*p*-value = 0.01).

For each symptom investigated (Table 1 summarizes the symptoms reported), a significant difference emerged among Wilayas for “somatic complaints” (*p*-value = 0.03); the post hoc test showed a significant difference between Laayoune (2.16% [95% CI: 1.56–2.91%]) and Auserd (0.14% [95% CI: 0.003–0.79%]) (*p*-value = 0.04).

**Figure 3:**
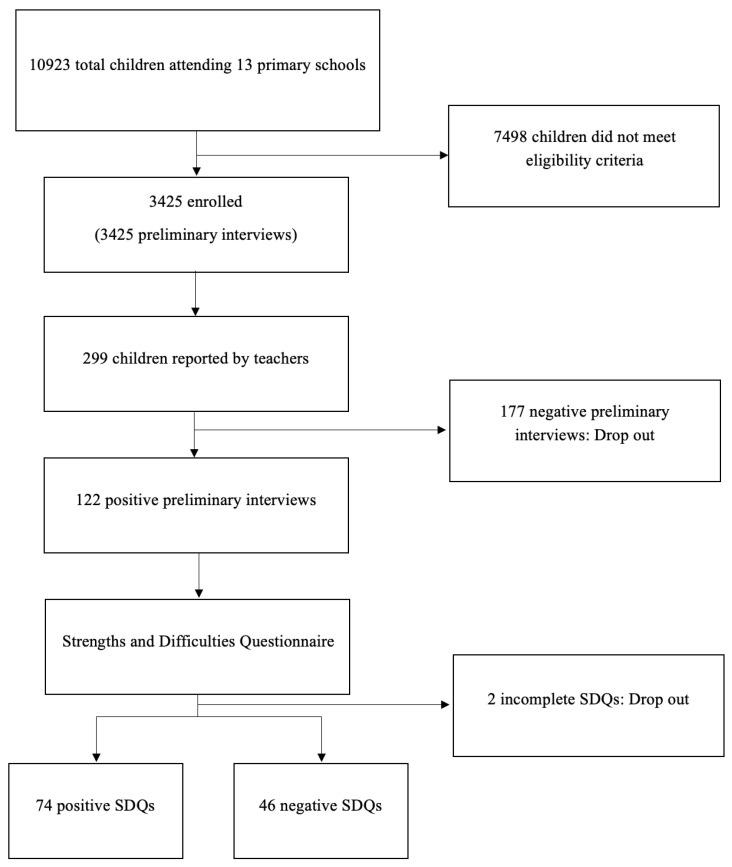
General overview of this study based on the STROBE flow diagram.

Out of the total 3425 students, 299 (8.7% [95% CI: 7.8–9.72%]) were reported by teachers in the preliminary interviews, but only 122 SDQs (3.5% [95% CI: 2.97–4.24%]) were completed. Of these, 2 SDQs (1.6% [95% CI: 0.2–5.8%]) were excluded since they were incomplete and 74 (2.2% of the total sample [95% CI: 1.7–2.7%] and 60.65% [95% CI: 51.4–69.4%] of the completed SDQs) had a positive total score. Additionally, 102 students (2.98% of the total sample [95% CI: 2.43–3.6%] and 83.6% [95% CI: 75.8–9.72%] of the completed SDQs) were positive on at least one subscale, though they did not necessarily exceed the cutoff for the total score. Figure 3 shows a general overview of this study based on the STROBE flow diagram [17].

### 3.3. Strengths and Difficulties Questionnaire (SDQ)

Out of a total of 122 completed SDQs, teachers filled in 26 SDQs (21.31% [95% CI: 14.42–29.65%]) in Smara, 23 SDQs (18.85% [95% CI: 12.34–26.93%]) in Auserd, and 73 SDQs (59.84% [95% CI: 50.6–68.6%]) in Laayoune. Most questionnaires were filled in for boys (94 SDQs; 77.05% [95% CI: 68.57–84.18%]) compared to girls (25 SDQ; 20.49% [95% CI: 13.72–28.75%]); for three questionnaires, sex was unknown (2.46% [95% CI: 0.51–7.02%]). Overall, 74 (2.2% of the total sample [95% CI: 1.7–2.7%] and 60.65% [95% CI: 51.4–69.4%] of the completed SDQs) SDQs were positive on at least one scale; the most common positive scales were conduct problems (48.36% [95% CI: 39.2–57.6%]), emotional problems (43.44% [95% CI: 34.5–52.7%]), and peer problems (40.16% [95% CI: 31.39–49.42%]). Table 2 summarizes the results.

Using ANOVA, the scores of the five scales and the total score were compared between the three Wilayas, and no statistical difference was found. The same result was found comparing the scores among the three different classes. The Student’s *t*-test comparing the scores on the hyperactivity scales between boys and girls revealed a significant difference between sexes, with a higher mean response in boys (*p*-value = 0.02). There were no significant sex differences on other scales or in the total score.

## 4. Discussion

Out of a total of 3425 children screened, 2.2% [95% CI: 1.7–2.7%] (74 children) received a positive SDQ result, indicating significant neurodevelopmental or psychiatric difficulties that had not been diagnosed, treated, or supported adequately in school.

A deeper analysis of the 102 SDQs positive for at least one subscale revealed that 31 (30.4% [95% CI: 21.67–40.28%]) were positive on only one scale, 27 (26.5% [95% CI: 18.22–36.13%]) were positive on two scales, and 44 (43.1% [95% CI: 33.36–53.32%]) were positive on three or more scales. Notably, the emotional problems scale was often associated with the conduct problem scale, while the conduct problem scale frequently correlated with the hyperactivity scale. Additionally, positivity on the peer problem and prosocial scales suggests potential challenges with social skills and peer relationships. These findings suggest the possibility of undiagnosed autism spectrum disorder traits or internalizing disorders, which are typically more challenging to diagnose but can have a significant impact on the daily lives of children [27].

Among children positive on three scales, the most common association was between the emotional problem, conduct problem, and peer problem scales. These data corroborate the findings of other studies conducted in analogous contexts [28]. This suggests that approximately 60% (59.46% [95% CI: 47.41–70.73%]) of the screened children experienced significant impairments across multiple domains, including emotion, behavior, and relationships. Supporting this, we found a direct correlation between the number of positive clinical scales and the impact score on the SDQ, confirming a negative impact on the children’s lives. Such complexities have been identified in the literature as risk factors for developmental trajectories, leading to adolescent and adult psychiatric disorders [25].

A recent review of the literature indicates that the prevalence of mental health problems in children and adolescents residing in LMICs is approximately 10–20%, consistent with the observations in HICs [29]. However, given this study’s design, the data cannot be regarded as representative of the general population. The SDQ questionnaire was administered to a selected group of pupils with potential risk factors, which limits the generalizability of the findings. Nevertheless, the data obtained demonstrate the presence of pupils (2.2% of the sample) with a high probability of neuropsychiatric disorders (indicated by the positivity to the total score of the SDQ questionnaire) who have never been diagnosed and for whom no intervention has been provided.

In addition to fulfilling this study’s primary objective of data collection, our time spent in schools raised teachers’ awareness and improved their ability to identify the symptoms of neurodevelopmental and psychiatric disorders in children. This was reflected by the good alignment between the initial interviews conducted to identify children with significant difficulties and the SDQ scores.

### 4.1. Strengths of This Study

The primary strength of this study lies in its status as a pioneering investigation within this particular population, as it identified a significant proportion of children encountering challenges who were unidentified and had received insufficient support within their educational and life contexts. Consequently, it directs attention toward the crucial issue of mental health in childhood within low-resource settings. Furthermore, this study highlights the feasibility of using a simple, structured screening tool, such as the SDQ, to identify children at risk for neurodevelopmental and psychiatric conditions, even in contexts where specialized resources are limited. Moreover, the engagement of teachers as key informants not only facilitated the identification of difficulties but also contributed to raising awareness and enhancing early detection within educational institutions.

### 4.2. Limitations of This Study

We observed variability in the teachers’ availability, participation, and knowledge regarding neurodevelopmental and psychiatric disorders. This discrepancy may stem from the novelty of conducting an epidemiological study in this specific population, particularly concerning the topics addressed and the instruments used. Additionally, we encountered a cultural barrier during this study, as relying on an interpreter instead of a cultural mediator complicated communication. This gap was particularly evident in the interviews; when a common language, such as Spanish, was used, teachers showed greater willingness to share their experiences, concerns, and insights.

To enhance the robustness of our findings, it is crucial to expand the sample by including all children attending the schools in Sahrawi refugee camps, particularly in Dakhla, which is more remote and may present distinct environmental and sociodemographic conditions. This will also improve the sensitivity and specificity of the SDQ as a screening tool in this population.

### 4.3. Future Research Directions

For future research, integrating these findings with data from parent interviews and direct evaluations will likely improve the sensitivity and specificity of the results, thereby ensuring greater generalizability [30]. More comprehensive epidemiological studies estimating childhood and adolescent psychopathology prevalence could enhance mental health prevention and inform future policies [31,32,33]. Additionally, future mental health policies should consider the significant role that schools play, specifically in Sahrawi culture. Since primary school attendance is compulsory, teachers serve as important role models for children, in addition to their families [34]. This proactive approach enables tailored interventions that address the needs of children with developmental disorders, facilitating their integration and support in the educational environment. Recognizing difficulties can be an important intervention for families, while educational interventions can improve the confidence and skills of primary care professionals [29,35]. The future impact of this intervention is establishing a public mental health promotion program that could lead to significant savings in educational, healthcare, and justice services. Problematic behaviors can have long-term negative effects on mental health, social relationships, and daily life [36,37].

Training and supervising professionals, particularly those working in schools, can achieve two main objectives. Firstly, school staff with higher education can more easily recognize the minor symptoms of neurodevelopmental and psychiatric disorders, identify vulnerable children, and monitor their academic progress and interaction with peers. Secondly, training teachers to identify symptoms is not merely for the recognition but rather for the early detection of these symptoms to initiate timely rehabilitation programs within the school setting. This proactive approach enables tailored interventions that address the needs of children with developmental disorders, facilitating their integration and support in the educational environment.

## 5. Conclusions

In conclusion, this study emphasizes the prevalence of untreated childhood neurodevelopmental and psychiatric disorders and highlights the need to sustain empowerment projects to safeguard children’s and adolescents’ mental health in LMICs. It is imperative that screening be continued in schools not included in this project, as there may be significant variations based on geographical location or demographic characteristics.

The considerable number of children with significant neurodevelopmental and psychiatric difficulties who had not received diagnosis, treatment, or adequate educational support suggests the need for all professionals working with children to possess basic skills identifying and managing these difficulties [34,36,37,38,39].

It is crucial to highlight that, in the initial phase of this study, the screened children were not clinically diagnosed, primarily due to the stigma associated with diagnosing such disorders. This is a significant challenge in many LMICs, where individuals often find it difficult to tolerate and accept such diagnoses [40]. In the Sahrawi population, despite the importance the SADR attaches to the issue of mental health, encouraging the implementation of psychological support for children and adults as well as mental health screening activities in the camp’s population, stigma persists [41].

Even in this scenario, the population seems reluctant to accept a diagnosis, which can often be a hindrance. Hence, it is imperative to increase awareness among the general population to diminish the apprehension surrounding diagnosis, enhancing their inclusion and ensuring tailored support within the school setting.

## Figures and Tables

**Table 1:** List of symptoms reported during the preliminary interview.

Symptoms	Number of Reports	Percentage (of 3425)
Hyperactivity/attention difficulties	172	5.02% [95% CI: 4.3–5.8%]
Anger outburst	13	0.38% [95% CI: 0.2–0.64%]
Sad/unhappy	14	0.41% [95% CI: 0.22–0.68%]
Lonely	8	0.23% [95% CI: 0.1–0.46%]
Insecure/easily scared	41	1.20% [95% CI: 0.86–1.62%]
Frequent complaining of pains (somatization)	51	1.49% [95% CI: 7.8–9.72%]

**Table 2:** SDQ form results.

	Total Positive (of 122 SDQ)	Very High/Very Low	High/Low	Average Answer
Emotional problems scale	53 (43.44%) [95% CI: 34.5–52.7%]	33 (27.05%) [95% CI: 19.4–35.83%]	20 (16.39%) [95% CI: 10.3–24.2%]	Slightly Raised (4.29 ± 2.28 SD)
Conduct problems scale	59 (48.36%) [95% CI: 39.2–57.6%]	40 (32.79%) [95% CI: 24.56–41.87%]	19 (15.57%) [95% CI: 9.64–23.25%]	Slightly Raised (3.45 ± 2.39 SD)
Hyperactivity scale	39 (31.97%) [95% CI: 23.82–41.02%]	23 (18.85%) [95% CI: 12.34–26.93%]	16 (13.11%) [95% CI: 7.69–20.4%]	Slightly Raised (6.12 ± 2.50 SD)
Peer problems scale	49 (40.16%) [95% CI: 31.39–49.42%]	25 (20.49%) [95% CI: 13.72–28.75%]	24 (19.67%) [95% CI: 13.03–27.84%]	Slightly Raised (4.30 ± 1.68 SD)
Prosocial scale	38 (31.15%) [95% CI: 23.07–31.43%]	28 (22.95%) [95% CI: 15.82–31.43%]	10 (8.20%) [95% CI: 4–14.56%]	Slightly Raised (5.47 ± 2.58 SD)
Total difficulties score	74 (60.65%) [95% CI: 51.4–69.4%]	58 (47.54%) [95% CI: 38.43–56.78%]	16 (13.11%) [95% CI: 7.69–20.4%]	High (18.16 ± 6.06 SD)
Impact score	54 (44.26%) [95% CI: 35.28–53.53%]	35 (28.69%) [95% CI: 20.86–37.58%]	19 (15.57%) [95% CI: 9.64–23.25%]	Slightly Raised (1.77 ± 2.62 SD)

## Data Availability

Data are available upon reasonable request from the corresponding author.
